# *In Vivo* engineering of transgenic mice for systemic human neutralizing antibody production against staphylococcal enterotoxin B

**DOI:** 10.3389/fimmu.2025.1679421

**Published:** 2025-11-18

**Authors:** Zhiyang Jiang, Beichen Jia, Naijing Hu, Mengmeng Zhang, He Xiao, Guojiang Chen, Jijun Yu, Xinying Li, Beifen Shen, Jiannan Feng, Jing Wang

**Affiliations:** 1 Academy of Military Medical Sciences, Beijing, China; 2 State Key Laboratory of National Security Specially Needed Medicines, Beijing, China

**Keywords:** staphylococcal enterotoxin B (SEB), genome-engineered bioreactors, CRISPR/Cas9, human monoclonal antibody, genomic safe harbors (ROSA26/H11)

## Abstract

Transgenic animal bioreactors provide a complementary strategy to traditional mammalian cell culture systems for the production of therapeutic human monoclonal antibodies (mAbs). Here we present a CRISPR/Cas9-mediated breakthrough in creating two novel genetically engineered (GE) mouse models with species-specific chromosomal integration of human anti-staphylococcal enterotoxin B (SEB) mAb genes at either the *ROSA26* or *Hipp11* (*H11*) safe-harbor loci - evolutionarily conserved genomic safe harbors (GSH). These genetically optimized animals demonstrated broad tissue capability for glycosylation-competent human antibodies, achieving exceptional secretion levels reaching 208 mg/L in serum, 43 mg/L in mammary secretions, 24 mg/L in saliva on average. The transgenic lines maintained this antibody production stability for >140 weeks without compromising animal viability, while preserving germline transmission fidelity through six successive generations. Furthermore, the highly glycosylated human antibodies derived from these genetic engineered mice exhibited high binding affinity to SEB (K_D_=0.108 nM for *ROSA26*; 0.154 nM for *H11*), providing comprehensive protection against SEB intoxication *in vivo*. This study opens avenues for utilizing transgenic animal bioreactors for large-scale production of fully human antibodies or disease-resistant livestock in the foreseeable future.

## 1 Introduction

Recombinant monoclonal antibodies (mAbs) have demonstrated remarkable success in treating cancers, inflammatory disorders, and infectious diseases (1–3). The growing clinical demand for antibody-based drugs has driven the development of large-scale production systems. Currently, mammalian cell culture—especially Chinese hamster ovary (CHO) cells—remains the industry standard for commercial antibody manufacturing (4). However, CHO-based production is associated with high cost, labor-intensive procedures, and scalability limitations (5–9), creating an urgent need for more efficient and economical alternatives.

Transgenic animals have emerged as promising “bioreactors” for pharmaceutical protein production, offering both high yield and physiologically relevant post-translational modifications (8–10). Several transgenic animal-derived biopharmaceuticals have already been approved, including Atryn^®^ (recombinant antithrombin from goat milk) and Ruconest^®^ (C1-esterase inhibitor from rabbit milk) (5, 11–13). More recently, the expression of monoclonal antibodies has been reported in diverse transgenic species, such as anti-PD-1 mAbs in mouse mammary glands (14–20), such as such as anti-PD-1 mAbs in mouse mammary glands (14), anti-CD20 mAbs in cow milk (21), humanized anti-HER2 mAbs in chicken eggs (16). Despite these advances, current strategies predominantly rely on tissue-specific expression (e.g., mammary gland or egg white) (14, 18, 21). Such approaches suffer from limitations including intermittent secretion, gender dependency, and inefficient use of male animals. Furthermore, random genomic integration often results in unpredictable regulation, genetic instability, and variable expression levels (14, 18, 21, 22). Thus, developing site-specific integration strategies that enable systemic and stable antibody expression remains a critical challenge.

*Staphylococcus aureus* is an important zoonotic pathogen widely transmitted among humans, livestock, and poultry, posing major public health threats (23). Its staphylococcal enterotoxin B (SEB) is a heat-stable superantigen that persists in animal populations (e.g., associated with mastitis) and can enter the human food chain, leading to severe food poisoning outbreaks (24). In our previous work, we identified a potent human antibody, LXY (LXY-Ab), from a phage display library, which effectively neutralized SEB toxicity *in vivo* (25).

**Figure 1:**
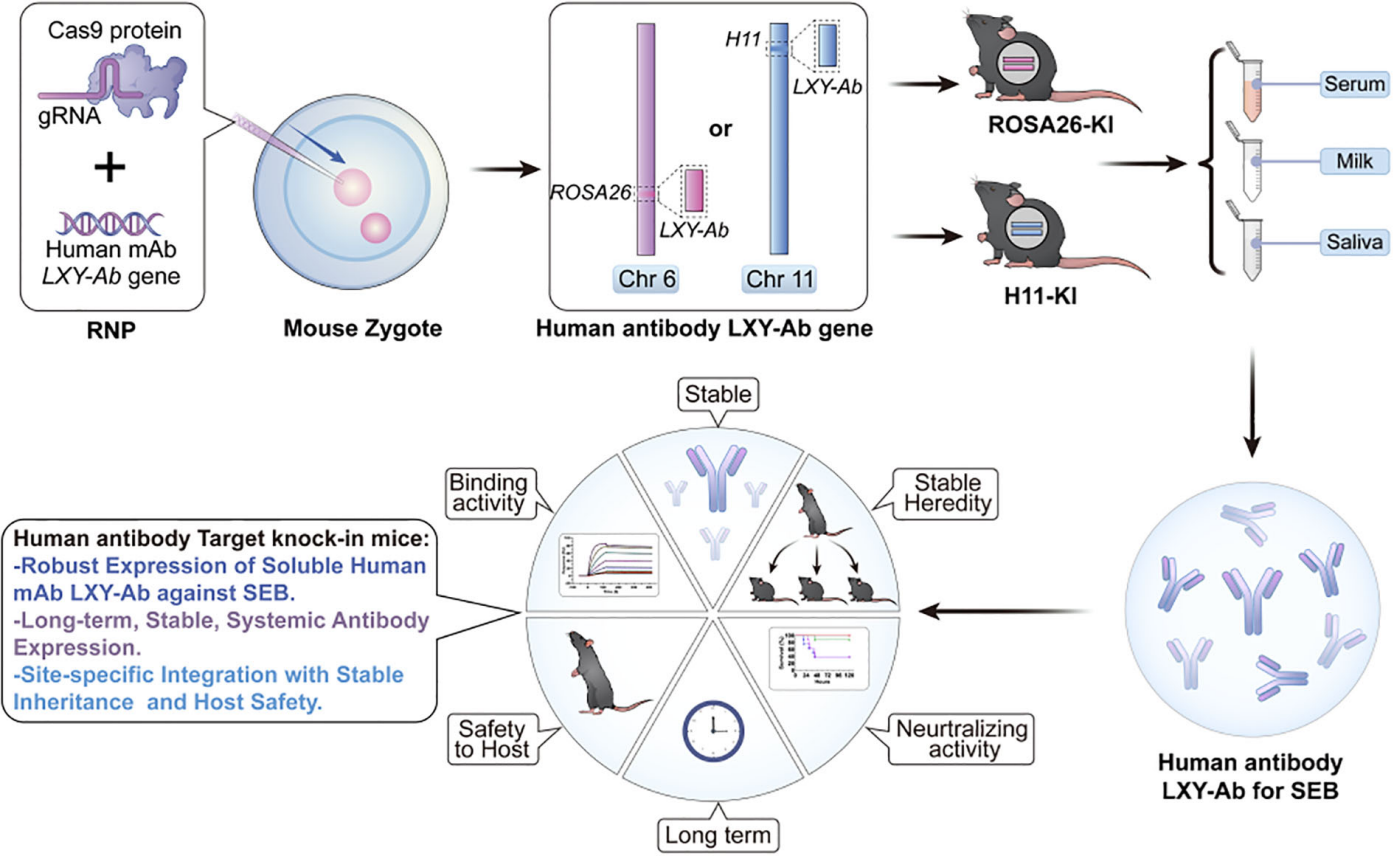
Locus-specific CRISPR/Cas9 integration enables stable and heritable production of human anti-SEB antibodies with preserved functionality in GE mice. This schematic depicts a genome-editing platform enabling independent integration of the human anti-staphylococcal enterotoxin B (SEB) monoclonal antibody LXY-Ab into two distinct genomic safe harbor loci: *ROSA26* (Chr6) or *H11* (Chr11). The strategy achieves site-specific, heritable insertion at either locus, ensuring robust and stable expression of fully glycosylated LXY-Ab across serum, milk and saliva. It maintains specific N-glycosylation patterns that preserve high SEB-binding affinity and *in vivo* neutralization efficacy. By facilitating comparative evaluation of *ROSA26*- and *H11*-targeted models, this platform combines locus-dependent expression stability with glycoform precision, advancing scalable *in vivo* production of therapeutic antibodies and serving as a robust tool for antitoxin development and functional validation.

In this study, we established a CRISPR/Cas9-mediated site-specific integration strategy to generate genetically engineered (GE) mice with the LXY-Ab heavy and light chain genes inserted into well-characterized genomic safe harbor loci, ROSA26 and Hipp11 (H11). CRISPR/Cas9 was selected over traditional random transgenesis and emerging tools such as prime editing due to its unique suitability for large-fragment knock-in in zygotes. While conventional transgenic methods often suffer from unpredictable integration sites and variable expression, CRISPR/Cas9 combined with homology-directed repair enables efficient and precise insertion of multi-kilobase antibody cassettes with stable germline transmission. The resulting GE mice exhibited systemic expression of glycosylation-competent human antibodies across multiple biofluids, including serum, milk, and saliva (**Figure 1**). Notably, the antibodies displayed enhanced binding affinity compared with their CHO-expressed counterparts and provided robust *in vivo* protection against SEB intoxication. These findings not only demonstrate the feasibility of systemic antibody production in transgenic animals, but also underscore the potential of CRISPR-guided genomic engineering as a scalable platform for next-generation biopharmaceutical manufacturing.

## 2 Materials and methods

### 2.1 Animals

Specific pathogen-free (SPF) female C57BL/6 mice (6–8 weeks old) were obtained from Cyagen Biosciences Inc (Suzhou, China) and age-matched BALB/c mice from Vital River Laboratories (Beijing, China). Mice were housed under SPF environmental condition and fed a normal chow diet and water. The animal experiments were conducted in accordance with national guidelines and were approved by the Institutional Animal Care and Use Committee of Academy of Military Medical Sciences (IACUC-DWZX-2021-621).

### 2.2 Targeted integration of SEB antibody expression cassettes into ROSA26 and H11 loci

For targeted integration of SEB monoclonal antibody expression cassettes, two sgRNAs were designed to target the first intron of *ROSA26* (sgRNA sequence: 5′-ACTCCAGTCTTTCTAGAAGA-TGG-3′) and *H11* (sgRNA sequence: 5′-GAACACTAGTGCACTTATCC-TGG-3′). The chemically synthesized sgRNAs were cloned into pX330-Cas9 vector via *BbsI* sites and validated by T7E1 mismatch cleavage assays (26, 27). We constructed two homology-directed repair (HDR) donor plasmids (pCAG-mAb-ROSA26 with 2.7/2.6 kb homology arms; pCAG-mAb-H11 with 3.0/2.4 kb arms) containing a CAG promoter-driven expression cassette with IRES-linked heavy and light chains. Targeted integration was achieved through DNA homology-directed repair. After linearizing the vectors, we co-microinjected donor plasmids with Cas9 protein and sgRNAs into C57BL/6 zygotes using an Eppendorf microinjection system (FemtoJet 4i, Hamburg, Germany), enabling targeted integration via homology-directed repair.

### 2.3 Generation and genomic validation of GE mice

C57BL/6 zygotes were co-microinjected with CRISPR/Cas9 vectors (pX330 system) and donor vector fragments linearized by restriction endonuclease W using an Eppendorf FemtoJet^®^ 4i micromanipulator (Hamburg, Germany). Following pronuclear microinjection, embryos were transferred into pseudopregnant females using standardized surgical protocols. Founder animals were genotyped via multiplex PCR with primers spanning integration junctions (**Supplementary Table S1**), followed by RT-qPCR quantification of LXY-Ab transcript levels across tissues. For Southern blot validation, genomic DNA from tail biopsies was digested with *SspI* (*ROSA26* locus) or *MfeI/SacI/BstEII* (*H11* locus) at 37°C for 16 hr. And then hybridized with a probe to the 5’HR and 3’HR. The expected band size for the targeted *ROSA26* allele was 9.1 kb and 9.8 kb, while for the *H11* allele was11.5 kb and 8.9kb.

### 2.4 Biofluid collection and processing

Serum was collected via retro-orbital venous puncture using heparinized capillary tubes, clotted at room temperature for 30 min, and centrifuged at 6,000 × *g* for 10 min. Milk was obtained from lactating mice (4–6-month-old) diluted 1:10 in Tris-buffered saline (25 mM Tris-HCl, 100 mM NaCl, pH 7.4), and clarified by centrifugation at 14,000 × *g* for 10 min (4°C) to isolate whey fractions. Saliva was induced via intraperitoneal injection of pilocarpine-HCl (0.5 mg/kg; Sigma-Aldrich, P6503), accumulated during 5–10 min of supine positioning, and collected with calibrated glass micropipettes. All biofluids were aliquoted and stored at −80°C within 2 h of collection.

### 2.5 Histological analysis of engineered mice expressing human antibody

Genetically engineered mice were euthanized, and tissues from nine major organs (myocardium, liver, spleen, lung, kidney, brain, thymus, small intestine, and large intestine) were dissected. Tissue samples were trimmed into 1 × 0.5 cm blocks, fixed in 4% paraformaldehyde solution for 24 h at 4°C, and subsequently dehydrated in 75% ethanol for histological processing and archival storage. Fixed tissues were embedded in paraffin, sectioned into 5-μm-thick slices using a Leica RM2235 rotary microtome (Leica Biosystems, Germany), and stained with hematoxylin and eosin (H&E). Histological slides were imaged using an Olympus VS200 slide scanner and analyzed with OlyVIA 3.1 software (Olympus Corporation, Tokyo, Japan).

### 2.6 Western blot analysis

Serum samples from GE mice were mixed with 5× Laemmli buffer under reducing or non-reducing conditions and denatured at 95°C for 5 minutes. Proteins were separated on 12% SDS-PAGE gels (Bio-Rad Mini-PROTEAN system) and transferred to PVDF membranes using an eBlot L1 transfer system (GenScript, Nanjing, China). Membranes were blocked with 5% non-fat dry milk in Tris-buffered saline containing 0.1% Tween-20 (TBST) for 1 hour at room temperature. Immunoblotting was performed with horseradish peroxidase (HRP) -conjugated goat anti-human IgG (1:1,000; Thermo Fisher Scientific, Cat# A18811) or goat anti-mouse IgG (1:1,000; Thermo Fisher Scientific, Cat# A16072) for 1 h at 4°C overnight. Chemiluminescent signals were detected using a ChemiScope 6100 imaging system (Clinx Science Instruments, Shanghai, China).

### 2.7 Expression and purification of recombinant SEB antigen

The SEB antigen was expressed and purified as previously described with modifications (25). Briefly, a C-terminal hexahistidine (6×His)-tagged SEB gene was cloned into the pET28a prokaryotic expression vector and transformed into *Escherichia coli* BL21(DE3) cells. Recombinant SEB expression was induced under standard conditions, followed by bacterial lysis and clarification via centrifugation. The soluble fraction was subjected to immobilized metal affinity chromatography using Ni-NTA resin (Cytiva, Cat# 11003399) under native conditions. Purified SEB was dialyzed into PBS (pH 7.4) and quantified by UV absorbance. Final purity (>95%) was verified by Coomassie Brilliant Blue-stained SDS-PAGE (**Supplementary Figure S3**).

### 2.8 Binding kinetics analysis of SEB-specific lgGs via surface plasmon resonance spectroscopy

Binding kinetics of serum-derived SEB-specific IgGs were analyzed using a Biacore T200 SPR system (Cytiva, Uppsala, Sweden) equipped with CM5 sensor chips (Cytiva, Cat# BR100530). Anti-human Fc antibody (10 μg/mL in 10 mM sodium acetate, pH 5.0; Cytiva, Cat# BR100839) was immobilized via amine coupling chemistry using 0.4 M 1-ethyl-3-(3-dimethylaminopropyl) carbodiimide (EDC) and 0.1 M *N*-hydroxysuccinimide (NHS) for 7 min activation. Serum samples or LXY-CHO antibody were diluted 1:100 in HBS-EP+ buffer (10 mM HEPES, 150 mM NaCl, 3 mM EDTA, 0.05% surfactant P20, pH 7.4) and injected at 10 μL/min for 60 s to capture IgGs. SEB analyte (0.76–50 nM in HBS-EP+) was flowed at 30 μL/min with 120 s association and 300 s dissociation phases. Sensor surfaces were regenerated with 10 mM glycine-HCl (pH 1.5) for 30 s. Binding data were globally fitted to a 1:1 Langmuir interaction model using Biacore T200 Evaluation Software 3.1 (Cytiva), yielding association (K_on_), dissociation (K_off_) rate constants, and equilibrium dissociation constant (K_D_= K_off_/K_on_).

### 2.9 Quantification of human antibody in GE mouse biofluids via sandwich enzyme-linked immunosorbent assay (ELISA)

96-well plates were coated with goat anti-human IgG (1:500 in 0.1 M carbonate-bicarbonate buffer, pH 9.6; KPL, a SeraCare company, #01-10-06) overnight at 4°C. After blocking with 5% non-fat milk in PBS (1 h, 37°C), serially diluted human IgG1 standard (Cetuximab, Bristol-Myers Squibb) and pre-diluted biofluid samples (1:5 in PBS, 5-fold serial dilutions) were incubated for 1 h at 37°C. Plates were washed with PBST (PBS + 0.05% Tween-20), incubated with HRP-conjugated goat anti-human IgG (1:6,000; Thermo Fisher Scientific, #A18811; 30 min, 37°C), washed again, and developed with 3,3’,5,5’-tetramethylbenzidine (TMB) substrate (Thermo Fisher, Scientific, #34028; 15 min, dark). Reactions were stopped with 0.5 M H_2_SO_4_, and absorbance (450 nm) was measured using a SpectraMax M5 plate reader (Molecular Devices, San Jose, USA). Antibody concentrations were calculated from a 4-parameter logistic standard curve (R² > 0.99) using SoftMax Pro 7.0 software (Molecular Devices).

### 2.10 Detection of total mouse IgG in serum by sandwich ELISA

Total IgG levels in mouse serum were measured using an analogous sandwich ELISA. Briefly, 96-well plates were coated with goat anti-mouse IgG (Thermo Fisher Scientific, #A28174). After blocking, serial dilutions of a mouse IgG ELISA Standard (Thermo Fisher Scientific, # 39-50400-65) and diluted serum samples were applied. Bound IgG was detected using HRP-conjugated goat anti-mouse IgG (1:3,000; Thermo Fisher Scientific, #A16078), followed by TMB development and acid stop as above. Total IgG concentrations were determined against the mouse IgG standard curve.

### 2.11 Determination of antibody-SEB binding EC_50_ by ELISA

96-Microplates were coated with 2 μg/mL SEB in carbonate-bicarbonate buffer (pH 9.6) overnight at 4°C. After three washes with PBST, nonspecific binding was blocked with 5% skim milk in PBST for 1 h at 37°C. Two-fold serially diluted antibodies (starting at 1 μg/mL in PBST) were added in triplicate and incubated overnight at 4°C. Following washes, HRP-conjugated goat anti-human IgG (1:6,000 in PBST; Thermo Fisher Scientific, Cat# A18811) was added for 30 min at 37°C. TMB substrate was incubated for 15 min in the dark, reactions terminated with 0.5 M H_2_SO_4_, and absorbance measured at 450 nm. EC_50_ values were calculated using a four-parameter logistic model (GraphPad Prism v9.0).

### 2.12 Competitive ELISA for epitope analysis

SEB-coated plates (prepared as above described) were blocked with 5% skim milk in PBST. Serially diluted serum samples containing LXY-R26 or LXY-H11 (1:2 dilutions) were pre-incubated with biotinylated LXY-CHO (prepared using EZ-Link NHS-PEG4-Biotin, Thermo Fisher Scientific, Cat# A39259) for 30 min at 37°C. The pre-incubated mixtures were added to the SEB-coated plates in triplicate and incubated for 30 min at 37°C. After washing with PBST, streptavidin-HRP (1:3,000 dilution in PBST; Thermo Fisher Scientific, Cat# 21134) was added for 30 min at 37°C. TMB substrate was used for color development, and reactions were terminated with 0.5 M H_2_SO_4_ as described. Absorbance was measured at 450 nm. Competition binding curves were generated using GraphPad Prism v9.0.

### 2.13 Protective efficacy of recombinant LXY-Ab-containing serum in SEB-induced toxic shock

Female BALB/c mice (6–8 weeks old) were sensitized via intraperitoneal (i.p.) injection of D-galactosamine hydrochloride (D-GalN; 1 g/kg; Sigma-Aldrich, Cat# G0500). Thirty minutes post-sensitization, mice received i.p. injections of either SEB (0.25 mg/kg in PBS) or PBS (control). For therapeutic evaluation, a separate cohort was administered D-GalN (1 g/kg, i.p.) followed 30 min later by premixed solutions of SEB (0.25 mg/kg) with either LXY-Ab-containing transgenic serum (25 or 100 mg/kg LXY-Ab) or purified LXY-CHO antibody (100 mg/kg). Survival was monitored for 120 h post-injection, with mortality rates calculated at the study endpoint. Statistical significance was assessed using Kaplan-Meier survival analysis and log-rank test (GraphPad Prism v9.0).

### 2.14 N-glycan analysis of LXY10 mAbs from genetically engineered mice serum

Serum samples (GE mouse-derived or purified LXY-CHO) were thawed on ice, clarified by centrifugation (12,000 × *g*, 10 min, 4°C), and quantified via BCA assay (Pierce™, Cat# 23225). Proteins (50 μg/sample) underwent sequential reduction (5 mM DTT, 56°C, 30 min), alkylation (11 mM iodoacetamide, dark, 15 min), and tryptic digestion (1:50 w/w, 16 h, 37°C). Peptides were acetone-precipitated, reconstituted in 200 mM triethylammonium bicarbonate (TEAB) via ultrasonication, and enriched for glycopeptides using hydrophilic interaction liquid chromatography (HILIC) microcolumns with stepwise elution (0.1% TFA, 50 mM NH_4_HCO_3_, 50% acetonitrile). Enriched glycopeptides were separated on a C18 column (75 μm × 25 cm, 1.7 μm) via a Vanquish Neo UHPLC system with a 34-min gradient (4–99% acetonitrile/0.1% formic acid, 400 nL/min) and analyzed on an Orbitrap Astral mass spectrometer in data-dependent acquisition mode (full scan: 240,000 resolution at 700–2,000 *m/z*; MS/MS: 80,000 resolution; 0.6 s cycle time; 1,900 V nano-electrospray ionization).

Raw data were processed using Proteome Discoverer v3.0 and Byonic v4.0, searching human IgG1 Fc glycopeptides (UniProt: P01857) against the GlyGen database (https://www.glygen.org/). Glycans were classified into five subtypes (paucimannose, high mannose, complex/hybrid, fucosylated, sialylated) based on structural features (28), filtered by mass accuracy (≤5 ppm), Byonic score (≥300), and retention time (± 0.5 min). Site-specific co-glycosylation heterogeneity was quantified using Skyline v22.2 (MacCoss Lab, USA).

### 2.15 Data analysis

Data analyses employed parametric (Student’s *t*-test, two-way ANOVA with Tukey’s *post hoc* test) or non-parametric tests based on normality assessment (Shapiro-Wilk test, α=0.05). Categorical variables (e.g., phenotype frequencies) were analyzed using Chi-square/Fisher’s exact tests, while survival data utilized log-rank Mantel-Cox testing. Significance thresholds (^*^P<0.05, ^**^P<0.01, ^***^P<0.001, ^****^P< 0.0001) incorporated Bonferroni correction for multi-group comparisons. All analyses were conducted in GraphPad Prism 9.0 and R v4.2.1, with exact *P*-values and effect sizes reported where applicable.

## 3 Results

### 3.1 Generation and identification of GE mice targeting the ROSA26 and H11 loci

**Figure 2:**
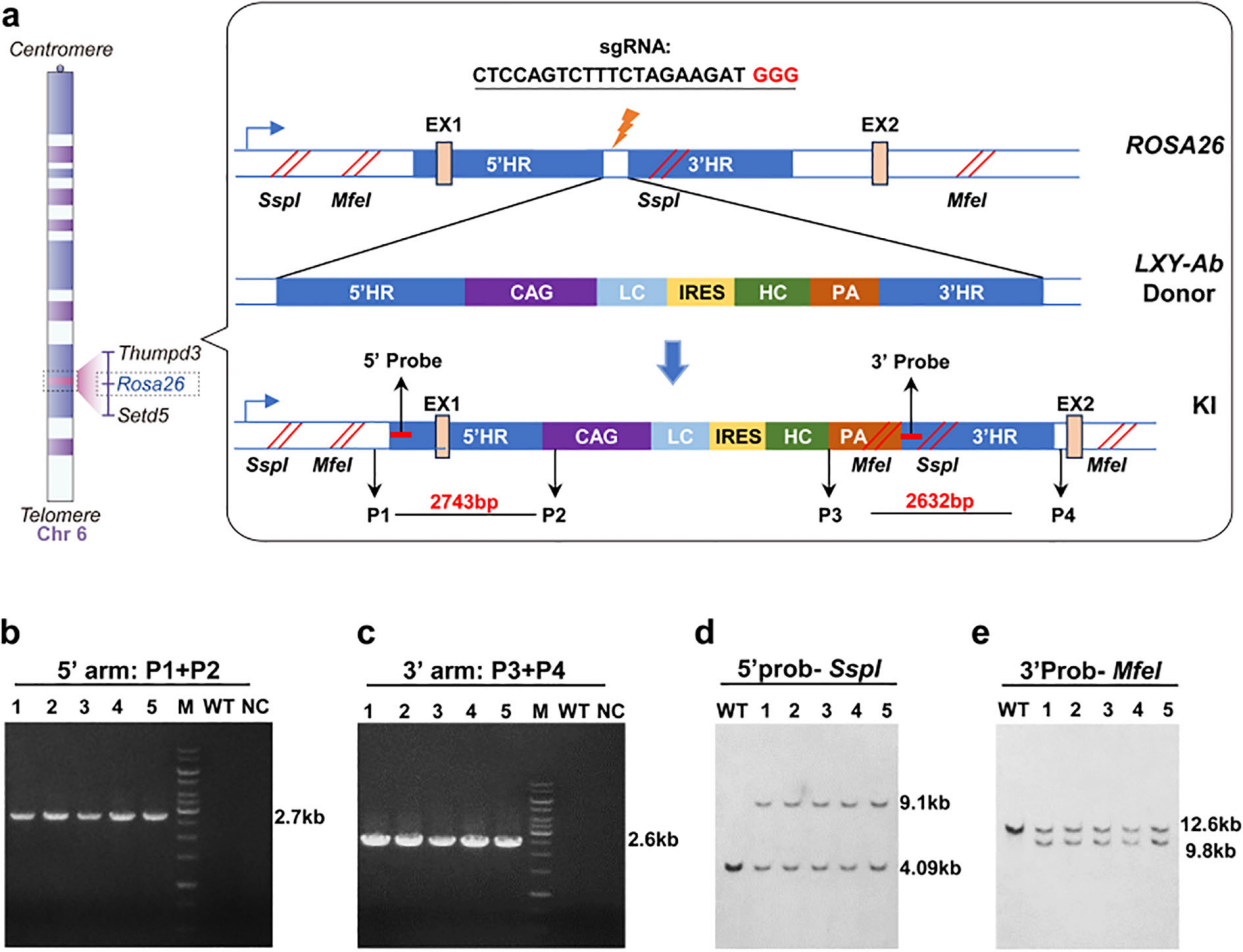
CRISPR/Cas9-mediated integration of human LXY-Ab at the *ROSA26* locus. **(a)** Schematic of the knock-in strategy targeting the first intron of the *ROSA26* locus (Chr6: 113,043,498–113,054,144). The donor vector contains 5′ and 3′ homology arms (5′HR: 2,115 bp; 3′HR: 2,153 bp), a CAG promoter-driven bicistronic cassette (light chain [LC] + IRES + heavy chain [HC]), a polyadenylation signal (PA), and restriction sites (*SspI*, *MfeI*) for Southern blot validation. **(b, c)** PCR genotyping of F1 GE mice using primers flanking the integration junctions, yielding 2.7 kb (5′) and 2.6 kb (3′) amplicons. Wild-type (WT) and no-template controls (NC) confirm specificity, with DNA ladder (15 kb marker, M) as reference. **(d, e)** Southern blot analysis of genomic DNA digested with restriction enzymes, hybridized with locus-specific probes. WT alleles display bands at 9.1 kb (*SspI*) and 12.6 kb (*MfeI*), while knock-in (KI) alleles yield 4.09 kb (*SspI*) and 9.8 kb (*MfeI*), confirming precise integration. Primer and probe sequences are detailed in **Supplementary Table S1**.

**Figure 3:**
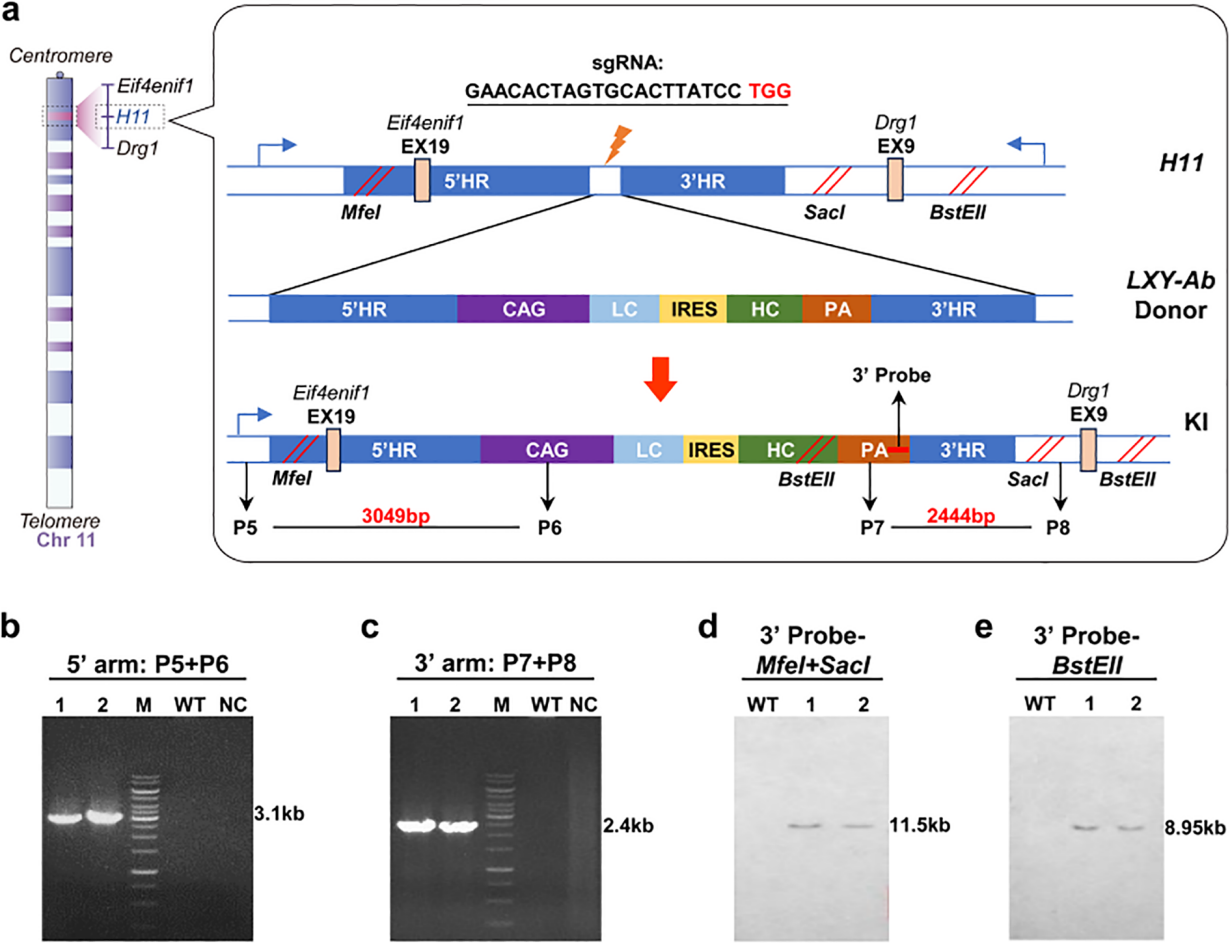
Construction and identifition of GE mice in *H11* locus. **(a)** Schematic of the *H11* locus (Chr11:94,129,124-94,138,327) knock-in strategy, targeting its first intron with a donor vector designed with 5′ and 3′ homology arms (2,104 bp and 2,038 bp, respectively), a CAG promoter-driven LC-IRES-HC bicistronic cassette, and a PA. Restriction sites (*MfeI*/*SacI, BstEII*) enable Southern blot validation. **(b, c)** PCR genotyping of F1 mice using locus-specific primers generates 3.1 kb (5′ junction) and 2.4 kb (3′ junction) amplicons, WT and no-template controls (NC) confirming specificity (15 kb DNA ladder, M). **(d, e)** Southern blot analysis using locus-specific probes distinguishes WT alleles (12.3 kb and 9.6 kb) from knock-in (KI) alleles (7.2 kb and 5.8 kb) following restriction enzyme digestion (*MfeI*/*SacI* for 5′ probe; *BstEII* for 3′ probe), validating precise integration. Primer/probe details are provided in **Supplementary Table S1**.

To enable site-specific expression of human anti-SEB antibody (LXY-Ab) in mouse, we engineered CRISPR/Cas9 knock-in systems targeting the *ROSA26* and *H11* genomic safe harbor loci. Donor plasmids pCAG-mAb-ROSA26 and pCAG-mAb-H11 incorporated locus-specific homology arms (5’HR/3’HR) to ensure targeted integration, a constitutive CAG promoter to drive antibody expression, bicistronic organization of light/heavy chain sequences via IRES-mediated co-expression, and transcriptional termination through a polyadenylation signal (**Figures 2a**, **3a**). Complementary CRISPR vectors (pX330-ROSA26Tg1 and pX330-H11Tg1) delivered locus-specific sgRNAs (26, 27) were engineered for precise double-strand break generation, Cas9 nuclease for homology-directed repair. Primers P1-P8 was designed to detect GE mice (**Supplementary Table S1**).

Pronuclear microinjection of paired donor/CRISPR systems into C57BL/6 mice zygotes yielded germline-transmissible integration efficiencies of 0.95% (*ROSA26*) and 1.30% (*H11*) (**Supplementary Table S2**), respectively. For *ROSA26* targeting, microinjection of 210 fertilized eggs into 8 pseudopregnant females produced 46 pups, with 2 transgenic founders confirmed by multiplex PCR (**Supplementary Table S2**). Similarly, *H11*-targeted microinjection of 230 eggs into 9 females generated 65 pups, identifying 3 transgenic founders. Founder (F0) mice were outcrossed with wild-type counterparts to eliminate mosaicism, followed by heterozygote intercrossing to establish homozygous F1 lines.

All F1 homozygotes underwent molecular validation: *ROSA26*-targeted lines exhibited 5’ (2.7 kb) and 3’ (2.6 kb) junctional PCR products (**Figures 2b, c**) and Southern blot fragments at 9.1 kb (*SspI*) and 9.8 kb (*MfeI*) (**Figures 2d, e**). *H11*-targeted lines showed 3.1 kb (5’) and 2.4 kb (3’) PCR amplicons (**Figures 3b, c**) with Southern blot bands at 11.5 kb (*MfeI/SacI*) and 8.9 kb (*BstEII*) (**Figures 3d, e**). Sanger sequencing of integration sites (**Supplementary Figure S1**) confirmed precise recombination. This tiered validation strategy—integrating fragment sizing, restriction mapping, and sequencing—definitively demonstrated locus-specific integration without detectable random events.

### 3.2 Characterization of GE mice expressing human antibody

**Figure 4:**
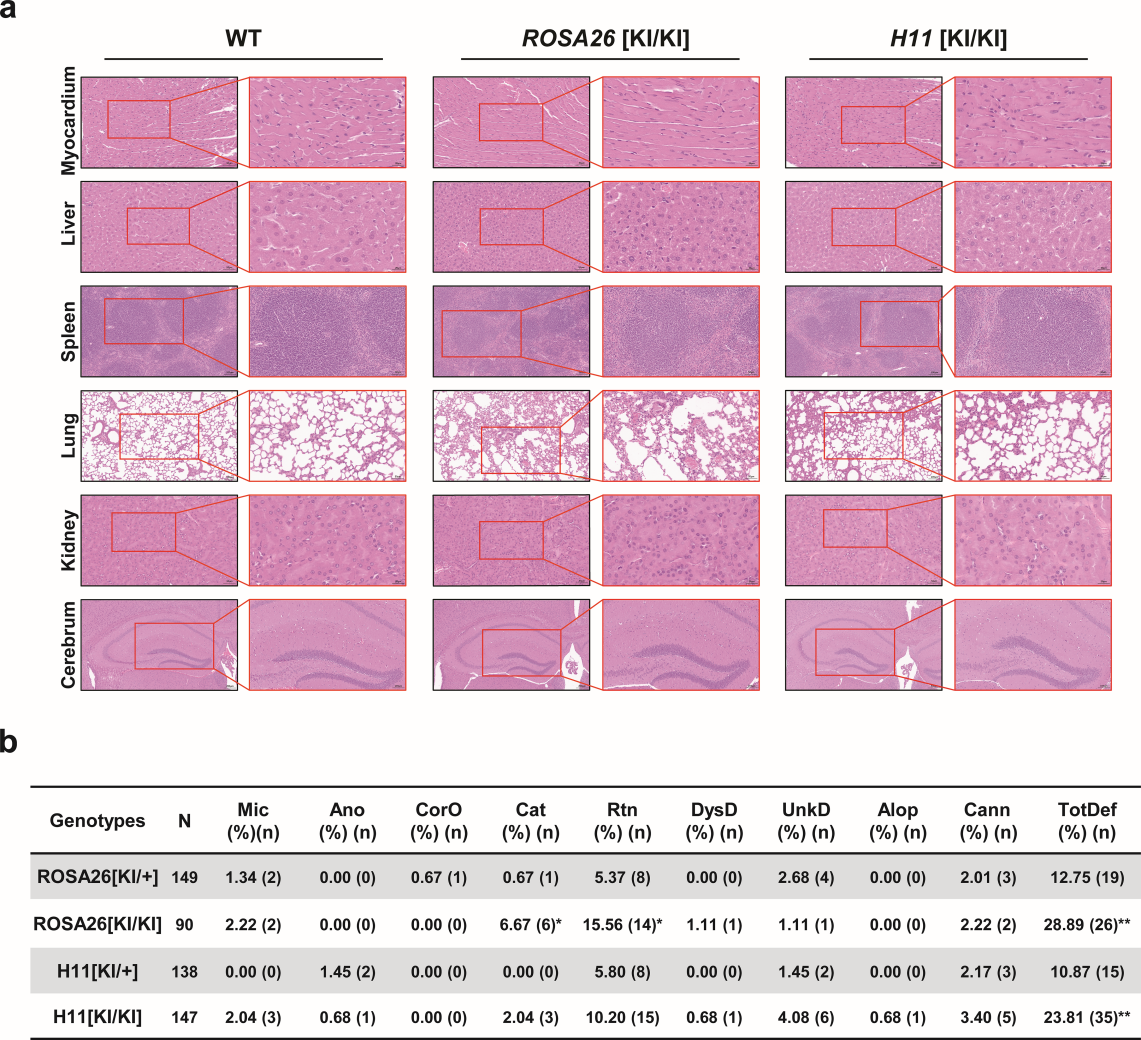
Histopathological and phenotypic assessment of ROSA26 and H11 knock-in mice. **(a)** Representative hematoxylin and eosin (H&E)-stained sections of myocardium, liver, spleen, lung, kidney, and cerebrum from wild-type (WT), *ROSA26* [KI/KI], and *H11* [KI/KI] mice. Tissues are displayed at two magnifications: spleen and lung (20×, scale bar: 20 μm; 40×, scale bar: 50 μm); myocardium, liver, kidney, and cerebrum (40×, scale bar: 20 μm; 100×, scale bar: 50 μm). Data represent three independent biological replicates. **(b)** Comprehensive phenotypic analysis comparing incidence rates (%) of developmental and behavioral phenotypes between heterozygous and homozygous mice: Microphthalmia (Mic), Anophthalmia (Ano), Corneal opacity (CorO), Cataract (Cat), Runting (Rtn), Dystocia-related death (DysD), Unexplained death (UnkD), Alopecia (Alop), Cannibalism (Cann), and Total defects (TotDef). Statistical significance (*P < 0.05, ** *P* < 0.01) was determined by χ² or Fisher’s exact test (applied when expected cell counts <5). Sample sizes: *ROSA26* [KI/+] (n=149), *ROSA26* [KI/KI] (n=90), *H11* [KI/+] (n=138), *H11* [KI/KI] (n=147).

Histopathological evaluation of hematoxylin and eosin (H&E)-stained tissues from *ROSA26*[KI/KI] and *H11*[KI/KI] antibody GE mice demonstrated preserved cytoarchitecture across major organs compared to wild-type (WT) controls (**Figure 4a**). Cardiomyocytes exhibited uniform alignment without hypertrophy or fibrosis, hepatic plates maintained normal lobular architecture without steatosis or inflammatory infiltrates, splenic follicles showed distinct red and white pulp demarcation, pulmonary alveoli had intact septal structures, renal glomeruli retained typical capillary tuft morphology, and cerebral cortical layers displayed orderly neuronal stratification. Supplementary tissues (**Supplementary Figure S2**), including the thymus and intestines, also revealed preserved corticomedullary organization and mucosal architecture, respectively. No genotype-specific pathologies—such as hyperplasia, metaplasia, or cellular degeneration—were observed, consistent with the established safety profiles of *ROSA26* and *H11* as genomic safe harbor loci (29).

Comparative phenotypic profiling of human antibody gene-targeted mouse models revealed locus- and genotype-dependent developmental variations across four transgenic lines (**Figure 4b**). While ocular anomalies in transgenic cohorts (2.7–8.9%) remained below the 12% spontaneous ocular defect rate in age-matched C57BL/6J WT mice (30). Notably, strain-specific anomalies such as hereditary hydrocephalus (1–4% in WT) and microphthalmia [Mouse Phenome Database (31)]—were absent across all transgenic lines. *ROSA26*-KI/KI mice exhibited dose-dependent increases in cataracts (6.67% vs. 0.67% in heterozygotes, *P* = 0.013) and growth retardation (15.56% vs. 5.37%, *P* = 0.028). Inter-locus comparisons demonstrated mutually exclusive phenotype clustering, with cataracts restricted to *ROSA26*-KI/KI and alopecia exclusive to *H11*-KI/KI, both lines maintaining stable germline transmission through six generations without phenotypic attenuation (data not shown). Collectively, these findings indicate that targeted human antibody *LXY-Ab* gene integration at *ROSA26* and *H11* loci does not globally compromise murine health beyond natural developmental variability.

### 3.3 Tissue-specific expression and structural integrity of full-human LXY-Ab antibody in GE mice

**Figure 5:**
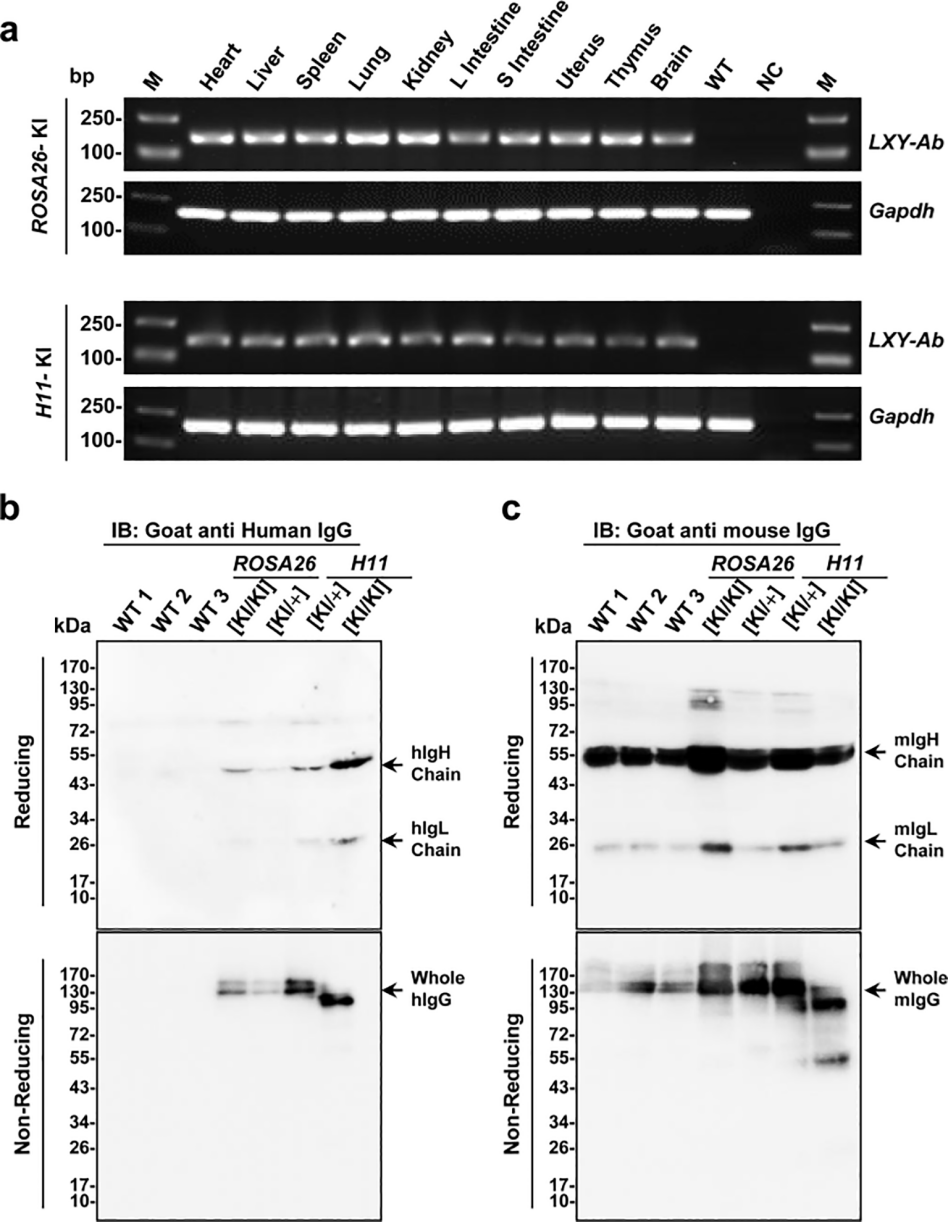
Tissue-specific expression and structural validation of full-human LXY-Ab in ROSA26 and H11 GE mice. **(a)** RT-PCR analysis of *LXY-Ab* mRNA expression across multiple tissues (heart, liver, spleen, lung, kidney, L intestine, S intestine, uterus, thymus, and brain) from *ROSA26* and *H11* homozygous knock-in mice. Amplification of the expected 180 bp product (*LXY-Ab*) and 150 bp GAPDH control confirmed tissue-wide transcription, with no signal in wild-type (WT) or no-template (NC) controls. M: DNA ladder. **(b)** Western blot under reducing conditions (goat anti-human IgG) detected LXY-Ab heavy chain (~55 kDa) and light chain (~25 kDa), with expression intensity correlating with zygosity (*KI/KI* > *KI/+*). Non-reducing conditions revealed intact IgG (~150 kDa). **(c)** Parallel blots probed with goat anti-mouse IgG verified endogenous murine IgG in all samples, excluding cross-reactivity. Molecular weight markers (kDa) are indicated. Each lane represents an independent biological replicate (WT1-3, *ROSA26* [KI/KI], *ROSA26* [KI/+I], *H11* [KI/KI] and *H11* [KI/+]).

RT-PCR and Western blot (WB) analyses were performed to evaluate the transcriptional and translational expression of the full-human LXY-Ab antibody in *ROSA26* and *H11* GE mice. Results (**Figure 5a**) show *LXY-Ab* transcription in multiple organs (heart, liver, spleen, lung, kidney, intestine, uterus, thymus, brain) in both loci, with no signal in wild-type controls, confirming *ROSA26* and *H11* as genomic safe harbors. Next, WB analysis (**Figure 5b**) shows revealed zygosity-dependent expression of LXY-Ab under reducing conditions, with distinct bands corresponding to the heavy chain (~55 kDa) and light chain (~26 kDa) showing significantly stronger intensities in homozygous ([KI/KI]) mice compared to heterozygotes ([KI/+]). Non-reducing conditions further confirmed the structural integrity of LXY-Ab, as evidenced by a single band at ~150 kDa corresponding to the fully assembled antibody (**Figure 5c**), probed with anti-mouse IgG, verifies endogenous murine IgG in all samples, excluding cross-reactivity. Collectively, these results confirm *ROSA26* and *H11* as robust loci for systemic, zygosity-dependent expression of full-human LXY-Ab in mice.

### 3.4 Expression of anti-SEB recombinant human antibodies in multiple biofluids of GE mice

**Figure 6:**
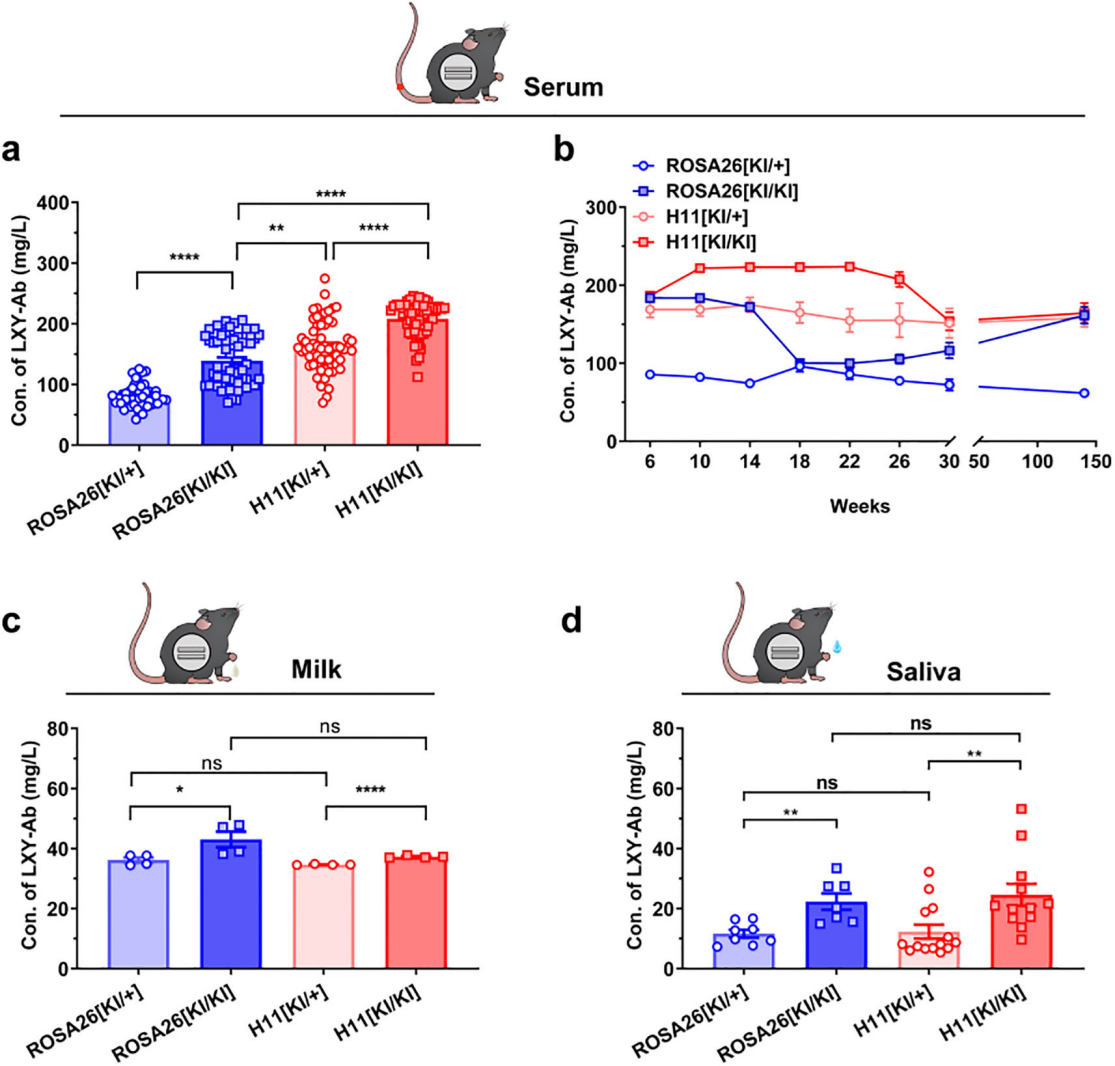
Systemic and sustained expression of SEB-specific monoclonal antibody LXY-Ab in GE mice. **(a)** Serum LXY-Ab concentrations in heterozygous (*ROSA26* [*KI/+*], *H11* [*KI/+*]) and homozygous (*ROSA* [*KI/KI*], *H11* [*KI/KI*]) mice quantified by ELISA (6–12 weeks, *n* = 53–61). **(b)** Age-dependent serum antibody dynamics over 6–140 weeks (*n* = 9/timepoint/genotype) **(c)** Postpartum day-7 milk in lactating females (*n* = 4/genotype) and **(d)** Stimulated saliva antibody concentrations across genotypes (*n* = 7–14). Data represent mean ± SEM. Statistical significance was determined by two-way ANOVA with Tukey’s *post hoc* test (^*^P<0.05, ^**^P<0.01, ^****^P< 0.0001; ns = not significant).

Longitudinal analysis of human antibody LXY-Ab expression revealed stable, tissue-specific production profiles across multiple biofluids in GE mice (**Figure 6**). The CAG promoter drove systemic antibody secretion in serum (**Figures 6a, b**), milk (**Figure 6c**), saliva (**Figure 6d**) with homozygous genotypes (KI/KI) universally outperforming heterozygotes (KI/+). In serum, *H11*[KI/KI] achieved peak concentrations (207.9 ± 33.5 mg/L, +31% vs *H11*[KI/+], P = 0.002; **Figure 6a**) and maintained stability over 140 weeks (<5% decline, **Figure 6b**). Tissue-specific dominance emerged: *H11*[KI/KI] dominated saliva (25.8 ± 3.6 mg/L, 2.3-fold over *H11*[KI/+], P = 0.002; **Figure 6d**), while *ROSA26*[KI/KI] excelled in milk (43.0 ± 2.3 mg/L, +94% vs *ROSA26*[KI/+], P < 0.0001; **Figure 6c**).

To assess the contribution of the transgenic antibody to the overall antibody pool, we calculated the proportion of LXY-Ab relative to the total serum IgG. The total mouse IgG concentration was found to be within the normal range of 3–5 g/L (32, 33), indicating no major perturbation of the endogenous immune system. Based on our measurements, LXY-Ab constituted approximately 2.9% to 4.4% of the total serum IgG across the different transgenic lines (**Supplementary Table S3**), confirming its robust and specific expression.

These results demonstrate that CAG promoter-driven targeted integration at *ROSA26* and *H11* loci supports robust, compartmentalized expression: *H11* favors systemic persistence (serum/saliva), whereas *ROSA26* enhances mucosal durability (milk). The sustained secretion over 140 weeks underscores these loci as genomic safe harbor loci for long-term human antibody production in mice.

### 3.5 Enhanced affinity and epitope conservation of recombinant anti-SEB antibodies from GE mice

**Figure 7:**
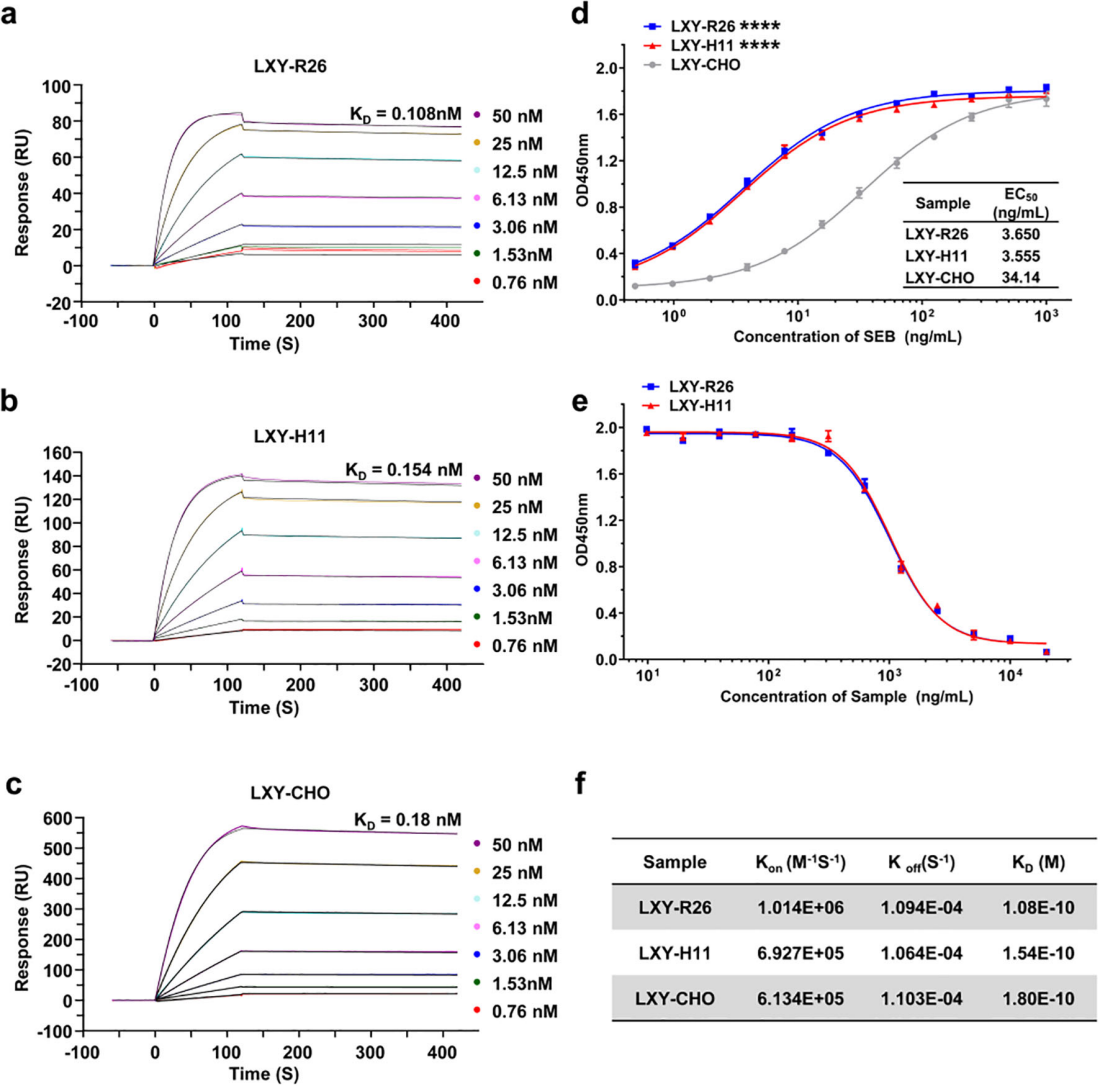
Functional characterization of LXY-Ab affinity and specificity across transgenic and CHO-derived sources. **(a–c)** Surface plasmon resonance (SPR) sensorgrams of SEB-binding kinetics for LXY-Ab derived from homozygous *ROSA26* [*KI/KI*] serum (LXY-R26, **(a)**, *H11* [*KI/KI*] serum (LXY-H11, **(b)**, and CHO-purified antibody (LXY-CHO, **(c)**. Anti-human Fc antibody was immobilized on a CM5 chip to capture LXY-R26, LXY-H11, or LXY-CHO, followed by SEB analyte injection (120 s association, 300 s dissociation). **(d)** ELISA dose-response curves binding to SEB comparing SEB-binding efficacy, with half-maximal effective concentrations (EC_50_) indicated (mean ± SEM, *n* = 3 independent replicates). **(e)** Competitive ELISA using biotinylated LXY-CHO, showing concentration-dependent inhibition by serum antibodies from both transgenic lines, confirming conserved epitope targeting. **(f)** Summary of kinetic parameters (K_on_, K_off_, K_D_) for all groups. ****P < 0.001 vs. LXY-CHO group.

Recombinant anti-SEB antibodies from *ROSA26* (LXY-R26, K_D_=0.108 nM) and *H11* (LXY-H11, K_D_=0.154 nM) GE mice exhibited 1.7- and 1.2-fold higher affinity, respectively, compared to CHO-expressed LXY-CHO (KD = 0.18 nM), as shown by surface plasmon resonance (SPR) (**Figures 7a-c, f**). The increased affinity was driven by accelerated association kinetics (LXY-R26: K_on_=1.01×10^6^ M^-1^s^-1^; LXY-H11: K_on_=6.93×10^5^ M^-1^s^-1^ vs LXY-CHO: K_on_=6.14×10^5^ M^-1^s^-1^). ELISA binding curves (**Figure 7d**) confirmed enhanced functional potency, with EC_50_ values for transgenic antibodies (3.65 and 3.55 ng/mL) 9.3-fold lower than LXY-CHO (34.14 ng/mL). Competition assays (**Figure 7e**) revealed complete (>99%) inhibition of LXY-CHO binding by both transgenic antibodies, confirming identical epitope specificity. These results demonstrate that the *ROSA26* and *H11* loci in mice enable production of fully human antibodies with superior affinity, accelerated antigen engagement, and unaltered epitope fidelity.

### 3.6 Glycosylation differences in GE mouse-derived LXY-Ab may contribute to enhanced SEB neutralization activity

**Figure 8:**
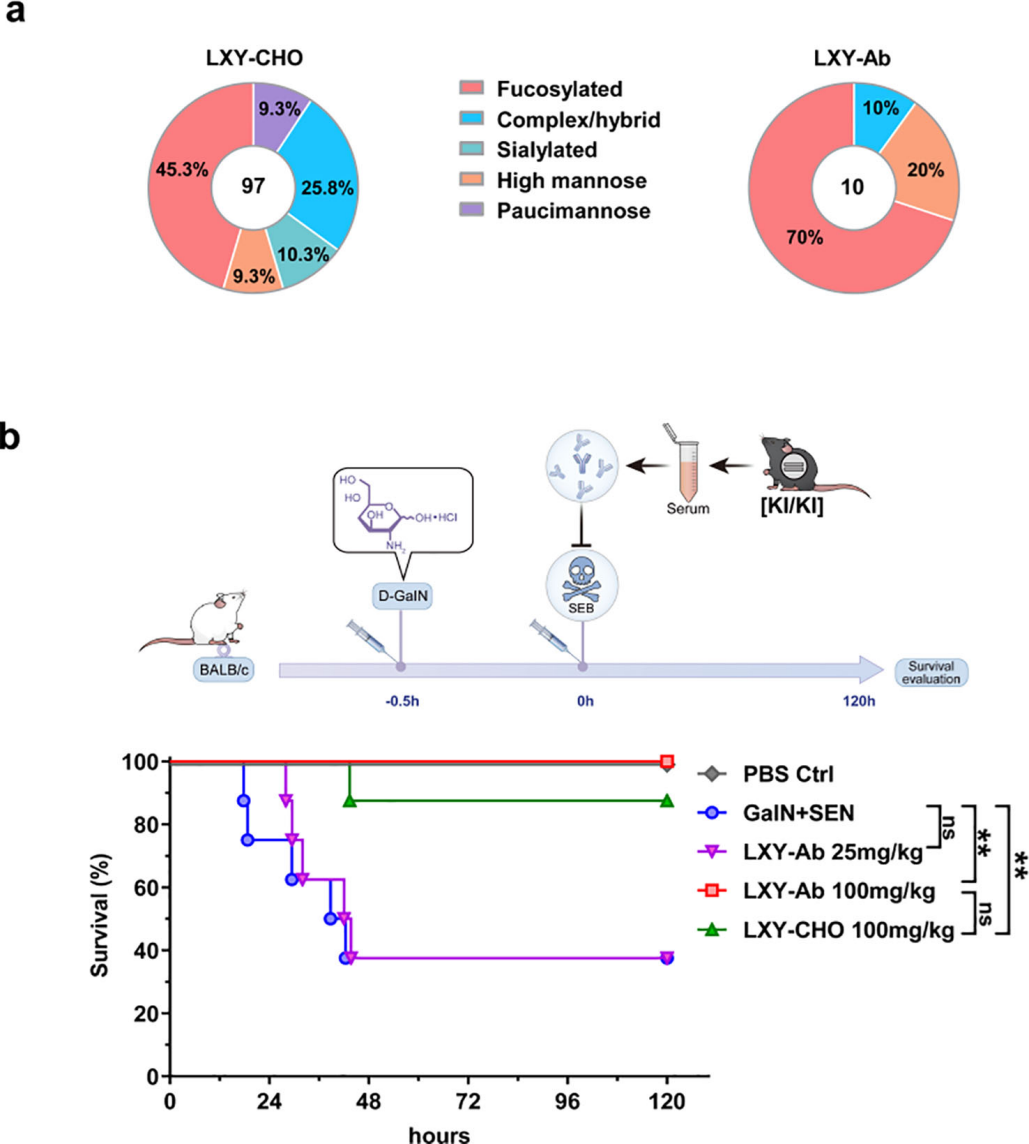
Glycan profiling and protective efficacy of GE mouse serum in SEB-induced toxic shock. **(a)** Comparative N-glycan profiling of LXY-Ab purified from GE mouse serum versus CHO cell-derived antibody. Percentages indicate the relative abundance of distinct glycoforms. **(b)** Schematic of the LD_50_ toxic shock model: BALB/c mice were sensitized with (D-GalN, 1 g/kg) followed by SEB (0.25 mg/kg). Survival kinetics of mice therapeutically treated (0 h) with transgenic serum [KI/KI] containing LXY-Ab (25 mg/kg, 100 mg/kg) or CHO-purified LXY-Ab (100 mg/kg). Untreated controls (D-GalN + SEB) and PBS-treated mice were included (n=8/group). Survival was monitored for 120 h post-SEB challenge. Data represent mean ± SEM from three independent experiments. Statistical significance was determined by log-rank test (**P<0.01, ns = not significant).

A comparative analysis of post-translational modifications (PTMs) in N-glycosylation patterns revealed expression system-dependent differences between CHO-derived LXY-CHO and GE mouse serum-expressed LXY-Ab (**Figure 8a**, **Supplementary Figure S4**). Liquid chromatography-mass spectrometry (LC-MS) profiling identified distinct glycoform distributions. LXY-CHO predominantly contained complex/hybrid glycans (25.8%) with moderate fucosylation (45.3%) and low sialylation (10.3%), while LXY-Ab exhibited enriched fucosylation (70%) and high-mannose glycans (20%), with undetectable sialylation. Co-occurrence network analysis further demonstrated greater glycan microheterogeneity in LXY-Ab compared to LXY-CHO (**Supplementary Figure S4**), likely attributable to species-specific glycosylation machinery in the GE mouse expression system. These differences in glycosylation profiles may impact the functional properties of the antibodies. Functional evaluation in a lethal SEB challenge model demonstrated dose-dependent protection (**Figure 8b**). Administration of 100 mg/kg LXY-Ab achieved complete survival (8/8, P<0.0001 vs. untreated controls), whereas the 25 mg/kg dose showed no significant benefit. Notably, the high-dose LXY-Ab exhibited a numerically superior survival rate compared to the CHO-expressed positive control LXY-CHO (100% vs. 87.5%, p=0.32), although statistical significance was not achieved. This trend may reflect enhanced affinity of the transgenic animal-derived antibody or differences in post-translational modifications between murine and CHO expression systems.

## 4 Discussion

The transgenic animal bioreactor system has emerged as a potential complementary platform for biopharmaceutical production, offering advantages over traditional Chinese hamster ovary (CHO) systems in cost-effectiveness, scalability, and eukaryotic post-translational modifications (PTMs) essential for therapeutic protein functionality (9, 34, 35). However, conventional approaches relying on tissue-specific promoters (e.g., mammary glands) limits scalability to female animals or random transgene integration face inherent limitations, including gender-restricted utility, regulatory instability, and low expression efficiency (9, 11, 36). Our study addresses these challenges through CRISPR/Cas9-mediated site-specific integration of anti-SEB antibody genes into *ROSA26* and *H11* genomic safe harbors (GSHs), achieving systemic and stable antibody expression in GE mice.

The selection of *ROSA26* and *H11* loci as integration sites was strategically motivated by their distinct advantages as GSHs. Both loci exhibit open chromatin structures conducive to stable transgene expression, with *ROSA26* demonstrating superior promoter accessibility across mammalian species and *H11* showing enhanced compatibility for large DNA fragment integration (37, 38). While our study achieved comparable integration efficiencies (0.95% vs 1.30%) and expression levels between these loci (**Figures 2**, **3**; **Supplementary Table S2**), *H11*-targeted constructs displayed marginally better expression consistency over successive generations (**Figure 6**), potentially attributable to its evolutionary conservation in transcriptional regulation. This systemic expression strategy circumvented the physiological interference concerns associated with ubiquitous transgene expression, as evidenced by normal murine development and histoarchitecture (**Figure 4**).

The bimodal distribution of LXY-Ab concentrations observed in *ROSA26*[KI/KI] mice (**Figure 6a**) suggests considerable individual variability in transgene expression. This heterogeneity may arise from post-transcriptional, post-translational, or epigenetic mechanisms. In particular, subtle epigenetic modifications—such as DNA methylation or histone acetylation at the *ROSA26* locus—could contribute to expression inconsistency, as previously reported for genomic safe harbor sites (39, 40). These results underscore the importance of accounting for individual variation in the breeding and evaluation of transgenic animal models. Future studies incorporating systematic epigenetic profiling will help clarify the regulatory mechanisms involved and improve the consistency of recombinant antibody production in transgenic bioreactor systems.

Notably, transgenic-derived LXY-Ab exhibited superior SEB-neutralizing efficacy compared to CHO-produced counterparts (100% vs 87.5% survival; *P* = 0.32; **Figure 8b**), potentially correlating with enhanced affinity (K_D_ = 1.08–1.54 nM vs. 1.8 nM; **Figure 6**) and unique N-glycosylation profiles (**Figure 8a**). Murine-derived antibodies displayed distinct PTM profiles characterized by enriched high-mannose glycans (20% vs CHO’s 9.3%) and elevated fucosylation (70% vs 45.3%) at Asn297 (**Figure 8a**). In comparison to human serum IgG, which typically exhibits core fucosylation at 90–96%, sialylation at 4–20%, high-mannose glycans at 1–2.5%, and dominant complex/hybrid glycans at 97.5–99% (41–43), the mouse-derived LXY-Ab shows fucosylation levels more aligned with human patterns than CHO-derived versions, though the elevated high-mannose content is atypical for circulating human IgG and may reflect species-specific cellular processing or purification challenges from mouse serum. Such species-specific PTM divergence may influence antibody affinity and effector functions (14, 43, 44), suggesting that transgenic systems could provide valuable platforms for generating “biobetters”.

Our platform advances transgenic bioreactor technology through three critical innovations: First, constitutive systemic expression via GSH integration resolves historical concerns about ectopic transgene toxicity while maintaining 140-week expression stability (**Figure 6**). Second, the multi-biofluid harvesting capability (serum: 82–208 mg/L; milk: 34–43 mg/L; saliva: 11–24 mg/L; **Figure 6**) overcomes the 50% biomass waste inherent in gender-restricted systems. Third, the CAG promoter-driven design ensures cross-tissue expression without mammary-specific purification challenges posed by casein interference. Although current titers (mg/L scale) trail mammary-specific systems achieving g/L outputs (21, 22). Thus, GE mice should be viewed primarily as a proof-of-concept research platform for rapid antibody prototyping and mechanistic studies. Future translation toward industrial application will likely require adaptation of this framework to larger livestock species, where volumetric yield and species-specific PTMs could be harnessed for scalable antibody production.

In summary, the human antibody target knock-in mouse bioreactors, characterized by robust human mAb expression, long - term, sustained systemic production, and stable heritable transmission, can be rapidly propagated. Our results also show that GE mice serum have better protective effect in a toxic shock syndrome model compared to mAbs derived from the traditional CHO cell culture systems. GE mice with systemic antibody expression represent a complementary strategy for producing recombinant mAbs. In principle, this strategy could be extended to antibodies targeting other bacterial toxins (e.g., anthrax toxin) or viral antigens (e.g., influenza hemagglutinin), which we plan to explore in future studies. The simple and feasible method provided in this study lays the foundation for the future large-scale production of fully human antibodies using transgenic animal bioreactors and offers new ideas for the preparation of disease-resistant livestock in the foreseeable future.

## 5 Conclusion

This proof-of-concept study establishes the biosafety of systemic expression of exogenous pathogen-targeting antibodies in GE mice, yet highlights key challenges for therapeutic translation. Current limitations in serum antibody purification (constrained by murine blood volume) and expression titers warrant technical innovations, including micro-scale analytical platforms and expression cassette optimization via dual-GSH targeting or copy number amplification. Although GE mice provide a valuable *in vivo* platform for the expression of fully human antibodies, their translational application remains limited by species-specific differences in immune context and by lower production yields compared to CHO systems. Future work will require rigorous safety evaluation and potential adaptation to larger livestock species to overcome these limitations and enable therapeutic scalability. These advances will catalyze therapeutic translation, positioning transgenic bioreactors as pillars of next-gen biomanufacturing.

## Data Availability

The original contributions presented in this study are publicly available. The mass spectrometry proteomics data have been deposited to the ProteomeXchange Consortium via the PRIDE partner repository with the dataset identifier PXD070488. All additional datasets generated for this study can be found in the article and its **Supplementary Material**.

## References

[1] Carter PJ Rajpal A . Designing antibodies as therapeutics. Cell. (2022) 185:2789–805. doi: 10.1016/j.cell.2022.05.029, PMID: 35868279

[2] Chan AC Carter PJ . Therapeutic antibodies for autoimmunity and inflammation. Nat Rev Immunol. (2010) 10:301–16. doi: 10.1038/nri2761, PMID: 20414204

[3] Pantaleo G Correia B Fenwick C Joo VS Perez L . Antibodies to combat viral infections: Development strategies and progress. Nat Rev Drug Discov. (2022) 21:676–96. doi: 10.1038/s41573-022-00495-3, PMID: 35725925 PMC9207876

[4] Kim JY Kim Y-G GMJAm L . Cho cells in biotechnology for production of recombinant proteins: Current state and further potential. Biotechnology. (2012) 93:917–30. doi: 10.1007/s00253-011-3758-5, PMID: 22159888

[5] Dyck MK Lacroix D Pothier F Sirard MA . Making recombinant proteins in animals–different systems, different applications. Trends Biotechnol. (2003) 21:394–9. doi: 10.1016/S0167-7799(03)00190-2, PMID: 12948672

[6] Durocher Y Butler M . Expression systems for therapeutic glycoprotein production. Curr Opin Biotechnol. (2009) 20:700–7. doi: 10.1016/j.copbio.2009.10.008, PMID: 19889531

[7] Kelley B Industrialization of mab production technology: The bioprocessing industry at a crossroads. mAbs. (2009) 1(5):443–52. doi: 10.4161/mabs.1.5.9448, PMID: 20065641 PMC2759494

[8] Houdebine LM . Production of pharmaceutical proteins by transgenic animals. Rev Sci Tech. (2018) 37:131–9. doi: 10.20506/rst.37.1.2746, PMID: 30209423

[9] Bertolini LR Meade H Lazzarotto CR Martins LT Tavares KC Bertolini M . The transgenic animal platform for biopharmaceutical production. Transgenic Res. (2016) 25:329–43. doi: 10.1007/s11248-016-9933-9, PMID: 26820414

[10] Dove A . Milking the genome for profit. Nat Biotechnol. (2000) 18:1045–8. doi: 10.1038/80231, PMID: 11017040

[11] Wang Y Zhao S Bai L Fan J Liu E . Expression systems and species used for transgenic animal bioreactors. BioMed Res Int. (2013) 2013:580463. doi: 10.1155/2013/580463, PMID: 23586046 PMC3613084

[12] Yan H Gong X Xu M Guo X Chen Y Xue Y . Production of biologically active human factor ix-fc fusion protein in the milk of transgenic mice. Biotechnol Lett. (2020) 42:717–26. doi: 10.1007/s10529-020-02808-1, PMID: 32002712

[13] Houdebine LM . Transgenic animal bioreactors. Transgenic Res. (2000) 9:305–20. doi: 10.1023/a:1008934912555, PMID: 11131009 PMC7089244

[14] Gong G Zhang W Xie L Xu L Han S Hu Y . Expression of a recombinant anti-programed cell death 1 antibody in the mammary gland of transgenic mice. Prep Biochem Biotechnol. (2021) 51:183–90. doi: 10.1080/10826068.2020.1805755, PMID: 32808868

[15] Zeng F Liao S Kuang Z Zhu Q Wei H Shi J . Genetically engineered pigs as efficient salivary gland bioreactors for production of therapeutically valuable human nerve growth factor. Cells. (2022) 11. doi: 10.3390/cells11152378, PMID: 35954224 PMC9368069

[16] Mukae T Okumura S Watanobe T Yoshii K Tagami T Oishi I . Production of recombinant monoclonal antibodies in the egg white of gene-targeted transgenic chickens. Genes (Basel). (2020) 12. doi: 10.3390/genes12010038, PMID: 33396657 PMC7823952

[17] Kim YM Park JS Kim SK Jung KM Hwang YS Han M . The transgenic chicken derived anti-cd20 monoclonal antibodies exhibits greater anti-cancer therapeutic potential with enhanced fc effector functions. Biomaterials. (2018) 167:58–68. doi: 10.1016/j.biomaterials.2018.03.021, PMID: 29554481

[18] Castilla J Pintado B Sola I Sanchez-Morgado JM Enjuanes L . Engineering passive immunity in transgenic mice secreting virus-neutralizing antibodies in milk. Nat Biotechnol. (1998) 16:349–54. doi: 10.1038/nbt0498-349, PMID: 9555725 PMC7097410

[19] Sola I Castilla J Pintado B Sanchez-Morgado JM Whitelaw CB Clark AJ . Transgenic mice secreting coronavirus neutralizing antibodies into the milk. J Virol. (1998) 72:3762–72. doi: 10.1128/JVI.72.5.3762-3772.1998, PMID: 9557658 PMC109598

[20] van Kuik-Romeijn P de Groot N Hooijberg E de Boer HA . Expression of a functional mouse-human chimeric anti-cd19 antibody in the milk of transgenic mice. Transgenic Res. (2000) 9:155–9. doi: 10.1023/a:1008987403484, PMID: 10951698 PMC7089348

[21] Zhang R Tang C Guo H Tang B Hou S Zhao L . A novel glycosylated anti-cd20 monoclonal antibody from transgenic cattle. Sci Rep. (2018) 8:13208. doi: 10.1038/s41598-018-31417-2, PMID: 30181542 PMC6123398

[22] Zhang R Cui D Wang H Li C Yao X Zhao Y . Functional recombinant human anti-hbv antibody expressed in milk of transgenic mice. Transgenic Res. (2012) 21:1085–91. doi: 10.1007/s11248-012-9589-z, PMID: 22286336

[23] Merz A Stephan R Johler S . Staphylococcus aureus isolates from goat and sheep milk seem to be closely related and differ from isolates detected from bovine milk. Front Microbiol. (2016) 7:319. doi: 10.3389/fmicb.2016.00319, PMID: 27014240 PMC4789554

[24] Krakauer T . Staphylococcal superantigens: Pyrogenic toxins induce toxic shock. Toxins (Basel). (2019) 11. doi: 10.3390/toxins11030178, PMID: 30909619 PMC6468478

[25] Hu N Qiao C Wang J Wang Z Li X Zhou L . Identification of a novel protective human monoclonal antibody, lxy8, that targets the key neutralizing epitopes of staphylococcal enterotoxin b. Biochem Biophys Res Commun. (2021) 549:120–7. doi: 10.1016/j.bbrc.2021.02.057, PMID: 33667709

[26] Chu VT Weber T Graf R Sommermann T Petsch K Sack U . Efficient generation of rosa26 knock-in mice using crispr/cas9 in c57bl/6 zygotes. BMC Biotechnol. (2016) 16:4. doi: 10.1186/s12896-016-0234-4, PMID: 26772810 PMC4715285

[27] Li YS Meng RR Chen X Shang CL Li HB Zhang TJ . Generation of h11-albumin-rtta transgenic mice: A tool for inducible gene expression in the liver. G3 (Bethesda). (2019) 9:591–9. doi: 10.1534/g3.118.200963, PMID: 30591434 PMC6385985

[28] Riley NM Hebert AS Westphall MS Coon JJ . Capturing site-specific heterogeneity with large-scale n-glycoproteome analysis. Nat Commun. (2019) 10:1311. doi: 10.1038/s41467-019-09222-w, PMID: 30899004 PMC6428843

[29] Yu Z Shi J Zhang J Wu Y Shen R Fei J . Comparative study on the expression characteristics of transgenes inserted into the gt(rosa)26sor and h11 loci in mice. Acta Biochim Biophys Sin (Shanghai). (2024) 56:1687–98. doi: 10.3724/abbs.2024081, PMID: 38752269 PMC11693873

[30] Simon MM Greenaway S White JK Fuchs H Gailus-Durner V Wells S . A comparative phenotypic and genomic analysis of c57bl/6j and c57bl/6n mouse strains. Genome Biol. (2013) 14:R82. doi: 10.1186/gb-2013-14-7-r82, PMID: 23902802 PMC4053787

[31] Bogue MA Grubb SC Walton DO Philip VM Kolishovski G Stearns T . Mouse phenome database: An integrative database and analysis suite for curated empirical phenotype data from laboratory mice. Nucleic Acids Res. (2018) 46:D843–50. doi: 10.1093/nar/gkx1082, PMID: 29136208 PMC5753241

[32] Klein-Schneegans AS Kuntz L Fonteneau P Loor F . Serum concentrations of igm, igg1, igg2b, igg3 and iga in c57bl/6 mice and their congenics at the lpr (lymphoproliferation) locus. J Autoimmun. (1989) 2:869–75. doi: 10.1016/0896-8411(89)90013-9, PMID: 2619870

[33] Sarvas HO Seppala IJ Tahtinen T Peterfy F Makela O . Mouse igg antibodies have subclass associated affinity differences. Mol Immunol. (1983) 20:239–46. doi: 10.1016/0161-5890(83)90062-7, PMID: 6865950

[34] Hryhorowicz M Lipinski D Hryhorowicz S Nowak-Terpilowska A Ryczek N Zeyland J . Application of genetically engineered pigs in biomedical research. Genes (Basel). (2020) 11. doi: 10.3390/genes11060670, PMID: 32575461 PMC7349405

[35] Khillan JS . Transgenic animals as bioreactors for expression of recombinant proteins. Methods Mol Biol. (1997) 63:327–42. doi: 10.1385/0-89603-481-X:327, PMID: 9113660

[36] He J Li X Luo D Zhang C Hu S Li X . A new animal bioreactor for producing pharmaceutical proteins. Acta Biochim Biophys Sin (Shanghai). (2014) 46:826–8. doi: 10.1093/abbs/gmu062, PMID: 25033830

[37] Browning J Rooney M Hams E Takahashi S Mizuno S Sugiyama F . Highly efficient crispr-targeting of the murine hipp11 intergenic region supports inducible human transgene expression. Mol Biol Rep. (2020) 47:1491–8. doi: 10.1007/s11033-019-05204-9, PMID: 31811500

[38] Tamura S Yasuoka Y Miura H Takahashi G Sato M Ohtsuka M . Thy1 promoter activity in the rosa26 locus in mice: Lessons from dre-rox conditional expression system. Exp Anim. (2020) 69:287–94. doi: 10.1538/expanim.20-0002, PMID: 32051391 PMC7445056

[39] Godecke N Zha L Spencer S Behme S Riemer P Rehli M . Controlled re-activation of epigenetically silenced tet promoter-driven transgene expression by targeted demethylation. Nucleic Acids Res. (2017) 45:e147. doi: 10.1093/nar/gkx601, PMID: 28934472 PMC5766184

[40] Cabrera A Edelstein HI Glykofrydis F Love KS Palacios S Tycko J . The sound of silence: Transgene silencing in mammalian cell engineering. Cell Syst. (2022) 13:950–73. doi: 10.1016/j.cels.2022.11.005, PMID: 36549273 PMC9880859

[41] Dekkers G Plomp R Koeleman CA Visser R von Horsten HH Sandig V . Multi-level glyco-engineering techniques to generate igg with defined fc-glycans. Sci Rep. (2016) 6:36964. doi: 10.1038/srep36964, PMID: 27872474 PMC5131652

[42] de Haan N Falck D Wuhrer M . Monitoring of immunoglobulin n- and o-glycosylation in health and disease. Glycobiology. (2020) 30:226–40. doi: 10.1093/glycob/cwz048, PMID: 31281930 PMC7225405

[43] Kanda Y Yamada T Mori K Okazaki A Inoue M Kitajima-Miyama K . Comparison of biological activity among nonfucosylated therapeutic igg1 antibodies with three different n-linked fc oligosaccharides: The high-mannose, hybrid, and complex types. Glycobiology. (2007) 17:104–18. doi: 10.1093/glycob/cwl057, PMID: 17012310

[44] Bryan L Clynes M Meleady P . The emerging role of cellular post-translational modifications in modulating growth and productivity of recombinant chinese hamster ovary cells. Biotechnol Adv. (2021) 49:107757. doi: 10.1016/j.bioteChadv.2021.107757, PMID: 33895332

