# Supplementary material for: In Vivo engineering of transgenic mice for systemic human neutralizing antibody production against staphylococcal enterotoxin B
Source: Front Immunol. 2025 Nov 18;16:1679421. doi: 10.3389/fimmu.2025.1679421 (PMC12669199; doi:10.3389/fimmu.2025.1679421)
Supplement: Supplementary file 3 [file SupplementaryFile1.pdf]

Supplymentary information

In Vivo Engineering of Transgenic Mice for Systemic Human Neutralizing Antibody Production Against Staphylococcal Enterotoxin B

Zhiyang Jiang[a,b], Beichen Jia[a,b], Naijing Hu[a,b], Mengmeng Zhang[a,b], He Xiao[a,b], Guojiang Chen[a,b], Jijun Yu[a,b], Xinying Li[a,b] Beifen Shen[a,b], Jiannan Feng[a,b,*], Jing Wang[a,b,*]

[a] Academy of Military Medical Sciences, Beijing 100850, China.

[b] State Key Laboratory of National Security Specially Needed Medicines, Beijing 100039, China.

[*] Corresponding authors.

E-mail address: jingw_biomed@163.com (J. Wang), fengjiannan1970@qq.com (J. Feng)

Supplymental Figures

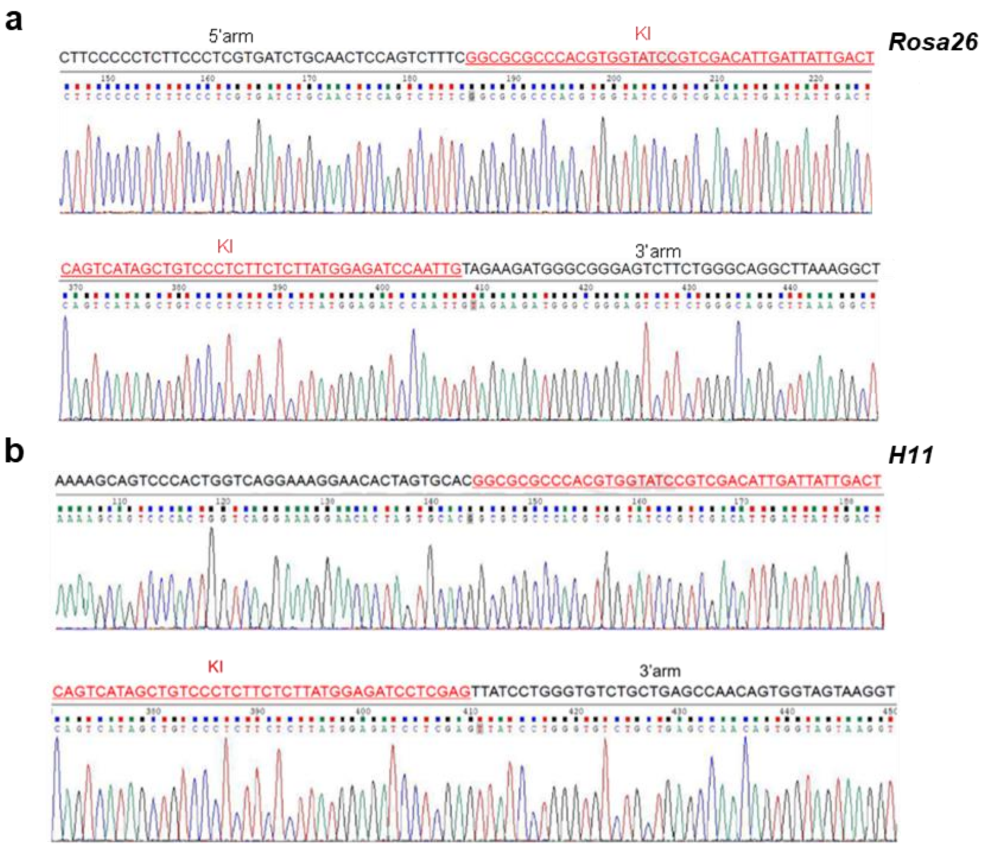

Fig. S1. Sanger sequencing validation of site-specific transgene integration at *ROSA26* and *H11* loci. Sanger sequencing confirmation of human antibody *LXY-Ab* gene knock-in (KI) at (a) *Rosa26* and (b) *H11* loci (n=3/locus). 5' and 3' junction PCR-amplified products from F1 homozygous mice were sequenced using primers spanning the donor plasmid-genome junctions (Table S1). No indels or off-target integrations were detected across all samples.

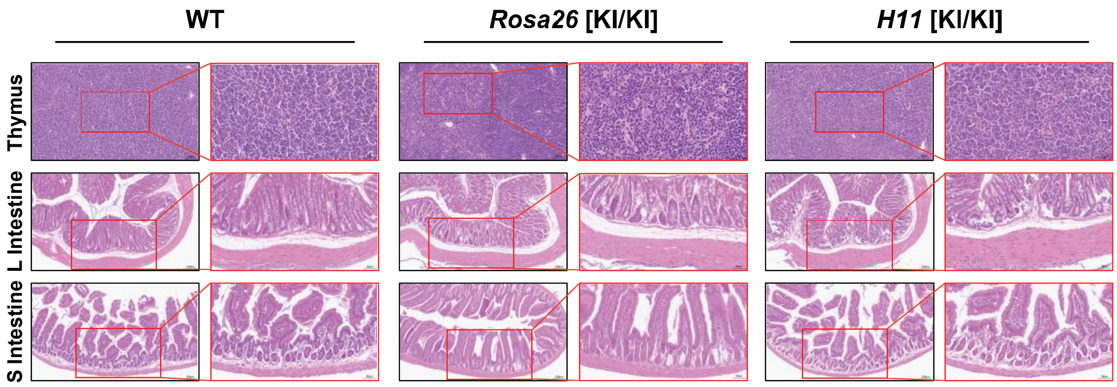

Fig. S2. Histopathological analysis of thymus, small intestine, and large intestine in LXY-Ab knock-in mice. Representative hematoxylin and eosin (H&E)-stained sections of thymus, small intestine (S Intestine), and large intestine (L Intestine) from 6-8-week-old wild-type (WT), ROSA26[KI/KI], and H11[KI/KI] mice. Tissues were analyzed for structural integrity of thymic corticomedullary architecture, small intestinal crypt-villus organization, and large intestinal mucosal morphology. Scale bar: 100 μm. No significant pathological alterations (e.g., inflammation, necrosis, or hyperplasia) were observed in transgenic lines compared to WT controls (n = 3 mice per group).

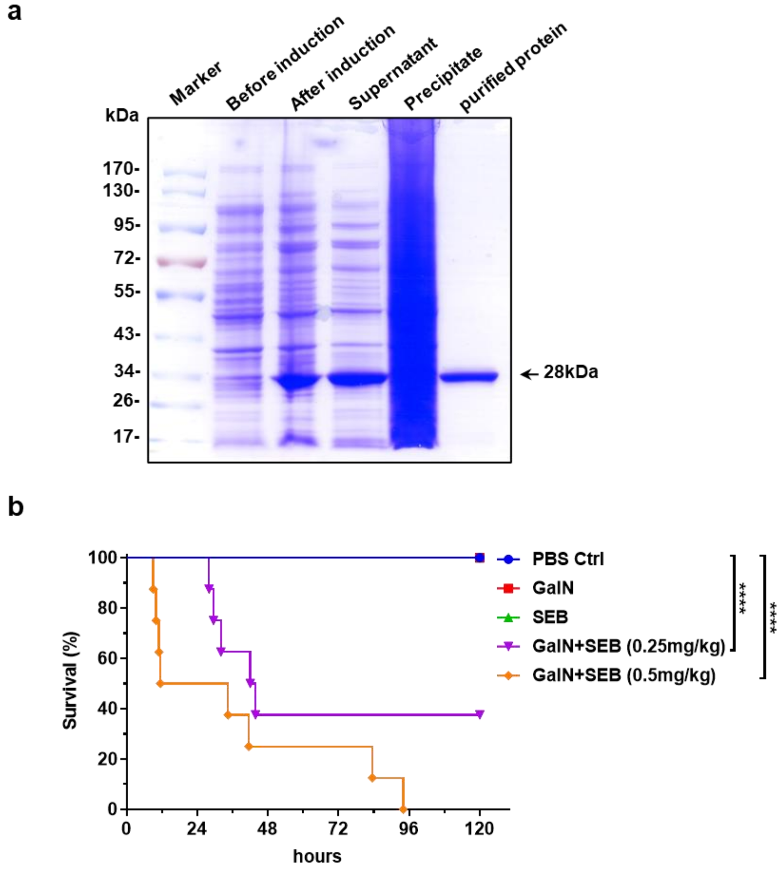

Fig. S3. Recombinant SEB production and validation of SEB-induced lethality. (a) Expression and purification of staphylococcal enterotoxin B (SEB) in *E. coli* BL21(DE3), analyzed by 12% SDS-PAGE. A 28 kDa band (arrowhead) corresponds to Ni-NTA affinity chromatography-purified SEB; protein standards (10–250 kDa) are labeled. (b) Survival kinetics of BALB/c mice (n = 8 per group) treated with: PBS (control), GalN (1 g/kg), SEB (0.5 mg/kg), GalN (1 g/kg) + SEB (0.25 mg/kg), or GalN (1 g/kg) + SEB (0.5 mg/kg). Mortality was monitored for 120 h post-injection, with ****P < 0.0001 (log-rank test) indicating significant survival advantage in PBS versus SEB groups.

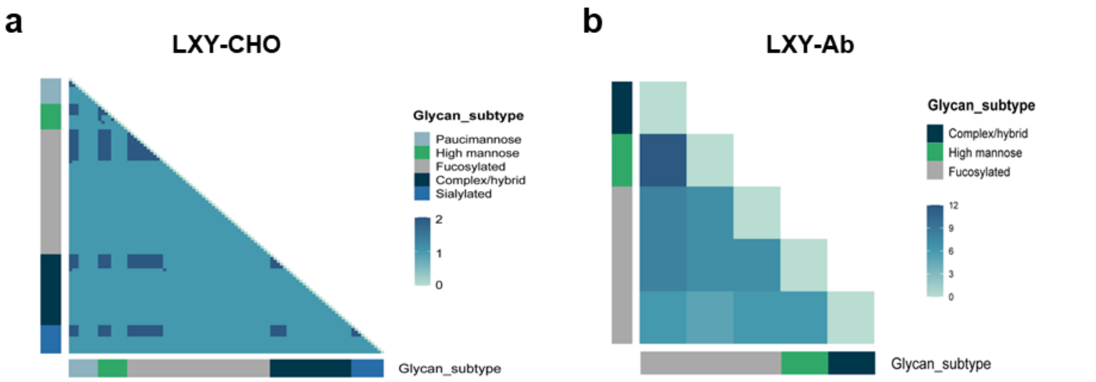

Fig. S4. Comparative analysis of N-glycosylation co-occurrence patterns in CHO-derived LXY-CHO versus transgenic mouse-derived LXY-Ab. Triangular heatmaps depict pairwise co-occurrence frequencies of five N-glycan subtypes (Pauximannose, High mannose, Fucosylated, Complex/hybrid, and Sialylated) in (a) CHO cell-derived LXY-CHO (scale: 0–2) and (b) transgenic mouse-derived LXY-Ab (scale: 0–12). Color intensity represents normalized co-occurrence frequencies (mean of three biological replicates).

Supplymental Tables

Table S1. Oligonucleotide primers or probe used in this study

Primer Name	Oligonucleotide Sequence (5′→3′)	Product Size (bp)	Purpose (Optional)
P1	AAAGATCGCTCTCCACGCCCTAG	2742	*ROSA26* 5'HR (F)
P2	AGATGTACTGCCAAGTAGGAAAGTC		*ROSA26* 5'HR (R)
P3	GCATCTGACTTCTGGCTAATAAAG	2632	*ROSA26* 3'HR (F)
P4	ATGGGAAGTTAGTAGCAAACAAGAG		*ROSA26* 3'HR (R)
P5	GTGGGCATTGGTTATTGGTCGTAG	3049	*H11* 5'HR (F)
P6	GATGGGGAGAGTGAAGCAGAACG		*H11* 5'HR (R)
P7	CTGCTGTCCATTCCTTATTCCATAG	2444	*H11* 3'HR (F)
P8	TGTGAGTCACCACGCTTGCCTTG		*H11* 3'HR (R)
P9	AGCCTCCTAAGCTCCTGATCT	180	qPCR LXY-Ab (F)
P10	GGGGGTAGGTGTAGTC		qPCR LXY-Ab (R)
P11	GGTTGTCTCCTGCGACTTCA	183	Gapdh qPCR (F)
P12	TGGTCCAGGGTTTCTTACTC		Gapdh qPCR (R)
Probe-1	AAACGTGGAGTAGGCAATACCCAGG	N/A	Southern blot *Rosa26* 5'probe (F)
Probe-2	AAAGAAGGGTCACCTCAGTCTCCCT		Southern blot *Rosa26* 5'probe (R)
Probe-3	TTCTGGGCAGGCTTAAAGGCTAAC	N/A	Southern blot *Rosa26* 3'probe (F)
Probe-4	AGGAGCGGGAGAAATGGATATGAAG		Southern blot *Rosa26* 3'probe (R)
Probe-5	TGCCCTGGCTCACAAATACCACT	N/A	Southern blot *H11* 3'probe (F)
Probe-6	TAGCCAACCTTTGTTCATGGCAGC-		Southern blot *H11* 3'probe (R)

Table S2. Targeted integration efficiency in transgenic mouse embryos

Group	Injected Eggs (n)	Survived Eggs (n)	Surrogate Mice Transplanted (n)	Pregnant Surrogates (n)	F0 Offspring (n)	Positive F0 Offspring (n)	TI Efficiency[b]
Rosa26	210	195	8	8	46	2 (TI) + 3 (RI)	0.95%
H11	230	210	9	8	65	3 (TI) + 2 (RI)	1.30%

[a]TI: Target integration; RI: Random integration.

[b]TI efficiency = (TI-positive F0 offspring / Injected eggs) × 100%

Table S3. Contribution of LXY-Ab to Total Serum IgG in Transgenic Mouse Lines

Genotype	Total Mouse IgG (g/L)	LXY-Ab (mg/L)	LXY-Ab / Total IgG (%)
ROSA26 [KI/+]	2.80 ± 0.10	81.80 ± 18.54	2.92 ± 0.66
ROSA26 [KI/KI]	3.95 ± 0.17	138.97 ± 40.77	3.52 ± 1.03
H11 [KI/+]	3.92 ± 0.90	163.44 ± 41.47	4.17 ± 1.28
H11 [KI/KI]	4.73 ± 0.12	207.90 ± 33.50	4.40 ± 0.71
